# Association between long-term static postures exposure and musculoskeletal disorders among university employees: A viewpoint of inflammatory pathways

**DOI:** 10.3389/fpubh.2022.1055374

**Published:** 2022-12-01

**Authors:** Yidan Dong, Ping Jiang, Xu Jin, Nanyu Jiang, Wenchu Huang, Yu Peng, Yuhong Shen, Lihua He, Mikael Forsman, Liyun Yang

**Affiliations:** ^1^Department of Occupational and Environmental Health, School of Public Health, Peking University, Beijing, China; ^2^Institute of Quartermaster Engineering & Technology, Beijing, China; ^3^Division of Ergonomics, School of Engineering Sciences in Chemistry, Biotechnology, and Health, Royal Institute of Technology, Huddinge, Sweden; ^4^Unit of Occupational Medicine, Institute of Environmental Medicine, Karolinska Institutet, Stockholm, Sweden

**Keywords:** musculoskeletal disorders, lower back pain, inflammatory cytokine, static postures, nested case-control study, university employees

## Abstract

**Background:**

Musculoskeletal disorders (MSDs) are critical occupational and social problems. With the improvement of production mechanization and automation, and the widespread application of computers, more occupations are exposed to static postures and load. This study explored the role of inflammation in the association between static postures exposure and MSDs.

**Methods:**

This study adopted a prospective nested case-control design in which 66 lower back MSDs cases and 66 healthy controls were selected from a cohort study of university employees. The personal information, postural load, musculoskeletal symptoms, pressure pain thresholds (PPTs), and inflammatory cytokines were collected. Logistic and linear regressions were used to investigate the association among postural load, inflammatory cytokines, and lower back MSDs. Mediation analysis was used to calculate the mediation effect.

**Results:**

The results of logistic and linear regressions showed that postural load and inflammatory cytokines were positively associated with lower back MSDs (*P* < 0.05), and postural load was positively associated with inflammatory cytokines (*P* < 0.05). Further, mediation analysis showed that the mediation effect of postural load on the lower back MSDs through TNF-α was 0.073 (95%CI: 0.025–0.128), and the mediation effect of posture load on the lower back MSDs through IL-6 was 0.098 (95%CI: 0.041–0.179), respectively.

**Conclusion:**

Static postures were associated with the occurrence of MSDs through inflammatory cytokines, and low-level inflammation may be a critical early event in the generation of MSDs. This study may help bridge the gap of potential mechanisms linking static postures to increased risks of MSDs, and provide new evidence for targeted protection against the global increasing MSDs.

## Introduction

Musculoskeletal disorders (MSDs) are major health problems globally (1). For example, data from the Global Burden of Disease Study 2019 showed that about 1.71 billion people worldwide suffer from MSDs, and MSDs are the highest contributors to years of life lived with disability (2). These diseases not only bring severe individual consequences such as disability, poor quality of life, and sickness absence but also impose a major economic burden on society (3). For example, the total cost of lost productivity attributable to MSDs was approximately 240 billion euros in the EU, up to 2% of the gross domestic product (GDP) (3).

Biomechanical factors (e.g., postural load) are considered as risk factors for MSDs (4). Static postures refer to physical exertion in which the same posture or position is held throughout the exertion. These types of exertions usually put a low level of sustained loads or forces on the muscles and tendons. Static postures mainly occur in occupational activities such as sitting, standing, prolonged bent or twisted posture of trunk, neck, or wrists, working with hands above shoulder level, kneeling or squatting posture (5). Prolonged postures can lead to static loading of the muscles and joint tissues and cause discomfort (6). Previous studies have shown that occupational activities requiring static loading of muscles and joint tissues constitute risk factors for the development of MSDs (7, 8). With the improvement of production mechanization and automation, as well as the widespread application of computers, more occupations are exposed to static postures and load (9, 10). Although the association between static postures and MSDs has been observed in humans, most studies are cross-sectional. There is a lack of human longitudinal studies on the causal relationship between static postures and MSDs.

Despite epidemiological evidence for the role of repetition, force, and postural load in the onset and progression of MSDs, complete understanding of these important health problems requires further elucidation of the pathophysiological mechanisms of MSDs. The National Occupational Research Agenda for Musculoskeletal Health emphasizes the need for etiologic research in determining the contribution of biomechanical mechanisms toward the development of tissue injury and MSDs, as well as strategies to reduce their severity (11). However, the mechanisms leading to pathophysiological tissue changes associated with MSDs are still incompletely understood.

The inflammation in musculoskeletal tissues may be an essential element in the pathogenesis of MSDs (12, 13). In a small number of studies, the severity of upper extremity MSDs has been positively associated with serum levels of tumor necrosis factor-alpha (TNF-α), interleukin-1alpha (IL-1α), interleukin-1beta (IL-1β) and interleukin- 6 (IL-6) in patients (14, 15). Freeland et al. have found higher levels of IL-6 and prostaglandin E_2_ (PGE_2_) in flexor tendon synovium in patients with carpal tunnel syndrome than in healthy controls (16). In addition, previous studies have also shown increased levels of TNF-α, IL-1α, IL-1β, and IL-6 in serum and muscle tissue in animals with injuries of the upper forelimb caused by excessive repetitive tasks (17, 18). The results of these studies suggested that inflammation may play an essential role in the course of MSDs. Therefore, it is plausible to hypothesize that static postures mediate or aggravate the occurrence of MSDs through inflammatory pathways. In light of the above findings, in this study, we aimed to explore the role of inflammation in the association between static postures exposure and MSDs based on a prospective nested case-control study.

## Materials and methods

### Study design

This population-based case-control study came from the cohort study of MSDs among university employees in a university in Beijing, China. This cohort study was conducted from March to August 2021. University employees usually work in a sitting position for a prolonged period. This cohort study recruited 400 healthy participants who had to meet the following criteria: (a) adults aged 18 to 30 years, (b) no history of smoking and drinking, (c) no history of musculoskeletal injuries, rheumatoid arthritis, tumors, tuberculosis, infections or other diseases affecting the musculoskeletal system, (d) no history of respiratory infections, nephritis, hepatitis, and other inflammatory diseases in the past 1 month, (e) no history of taking analgesic and anti-inflammatory drugs in the past 1 month, (f) no symptoms in the neck, shoulders, upper back, lower back, elbows, wrists/hands, hips/thighs, knees, and ankles/feet in the past 3 months. The baseline survey used questionnaires to collect information on general demographic characteristics, occupational characteristics, and musculoskeletal symptoms. In the baseline survey, 383 valid questionnaires were collected. Among them, 109 participants who withdrew from the study and those who did not meet the inclusion criteria of the case-control study were excluded, and 66 participants with lower back MSDs were included in the first follow-up survey 3 months later. Controls were randomly selected from the remaining healthy participants by 1-to-1 matching based on gender and age (±2 years). In this study, cases were taken from the participants who had lower back symptoms such as pain, discomfort, numbness, or limitation of movement in the past 1 month, which lasted for more than 24 h and had no relief after rest, without lower back symptoms caused by menstruation and exercise. Controls were selected from those who had no symptoms in the neck, shoulders, upper back, lower back, elbows, wrists/hands, hips/thighs, knees, and ankles/feet during the past 3 months.

**Figure 1:**
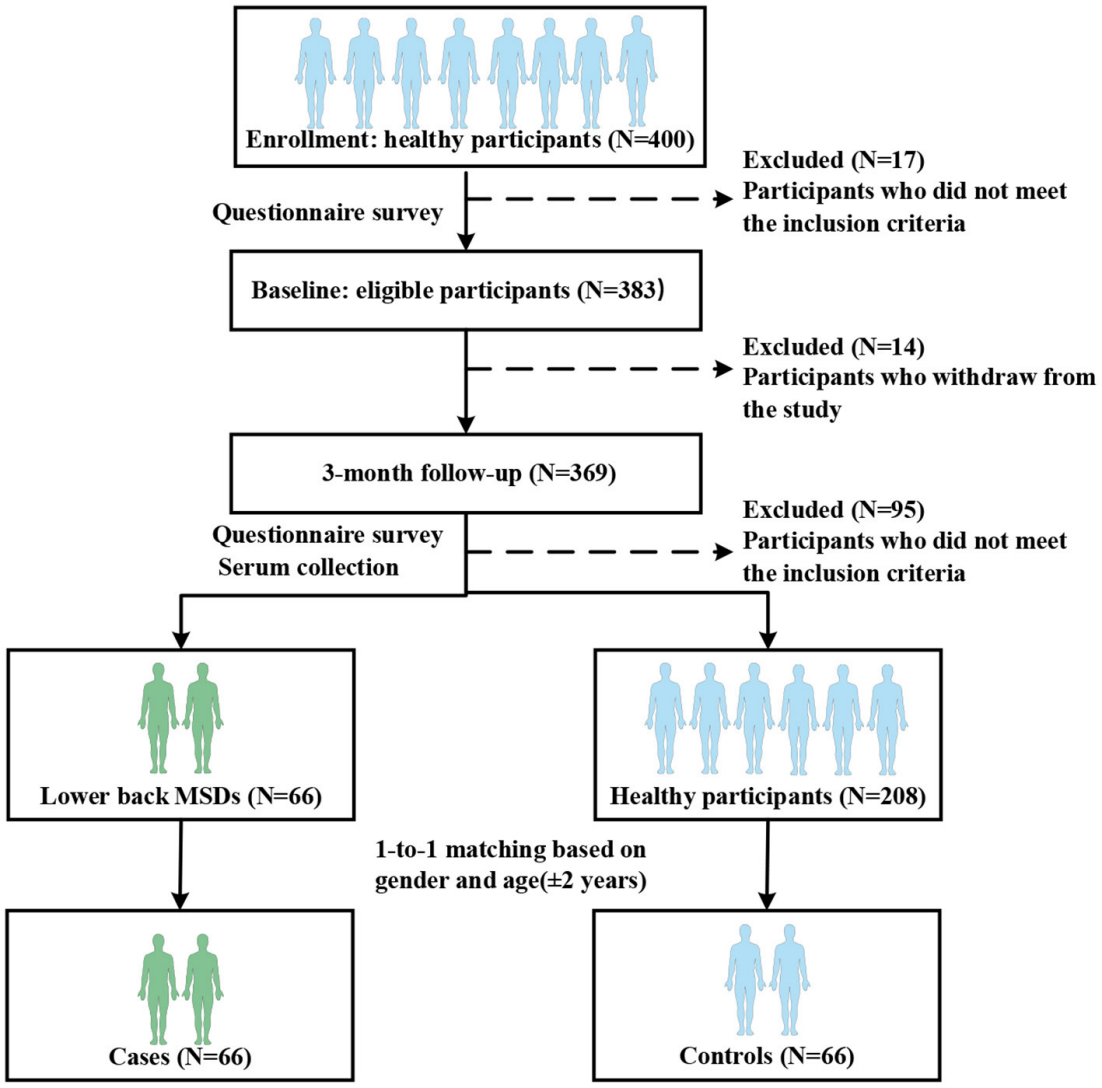
Flow chart of the nested case-control study.

In addition, cases and controls had to meet the following criteria during the follow-up period: (a) no history of smoking and drinking, (b) no history of musculoskeletal injuries, rheumatoid arthritis, tumors, tuberculosis, infections, or other diseases affecting the musculoskeletal system, (c) no history of respiratory infections, nephritis, hepatitis, and other inflammatory diseases, (d) no history of taking analgesic and anti-inflammatory drugs. Finally, 66 cases and 66 controls were included in this study. All participants provided written informed consent before the study began. This study was approved by the Institutional Review Board of Peking University (IRB0000105220087). The flow chart of the nested case-control study is shown in Figure 1.

### Data and sample collection

Information on demographic characteristics (gender, education, age, body mass index (BMI), and physical exercise time per week) and occupational exposure (hours spent per day working, hours spent per day working in a sitting position, hours spent per day using computers, hours spent per day using phones) were obtained through face to face interviews. The postural load of participants exposed to static postures was assessed by Rapid Upper Limb Assessment (RULA) (19). The overall Cronbach's α of RULA in this study was 0.764. RULA is divided into two sections labeled A and B. Section A covers the upper arms, lower arms, and wrists (upper limb score, 1–9 points), and section B covers the neck, trunk, and legs (lower limb score, 1–9 points). The scores for group A and group B postures and the scores for static muscle work and force are added to give an upper limb score and a lower limb score. The two scores are combined in a table to give a grand score (1–7 points). The English version of the instrument can be accessed by clicking on the following link: http://www.rula.co.uk. The grand score is divided into three levels, which are low postural load (1 to 4 points), moderate postural load (5 to 6 points), and high postural load (7 points).

The musculoskeletal symptoms were evaluated by the Nordic Musculoskeletal Questionnaire (NMQ) (20). The overall Cronbach's α in this study was 0.907. Participants were asked if they experienced musculoskeletal symptoms such as ache, pain, or discomfort during the past 1 month and during the past 3 months, by using a body map with nine body regions: neck, shoulders, upper back, low back, elbows, wrists/hands, hips/thighs, knees, and ankles/feet. Furthermore, symptoms in the past 1 month were assessed by self-reported pain duration (< 1 day, 1–7 days, 8–30 days, almost every day), pain frequency (1 time/month, 2–4 times/month, 5–10 times/month, >10 times/month, always symptomatic), and pain intensity (a 0 to 10 Visual Analog Scale (VAS): 0 mark as being painless, 1–3 marks as mild pain, 4–6 marks as moderate pain, 7–9 severe pain, and 10 marks as maximum pain) in each body part.

The pressure pain thresholds (PPTs) of the participants' lumbar multifidus muscles were measured by a hand-held digital force gauge (Wagner FDX 25, USA). The PPT was measured in all subjects in the prone position by trained physiotherapists. The force area of FDX 25 was a 1 cm^2^ round rubber tip, which was used to apply pressure to 2 cm lateral to the L4/L5 interspinous space (21). The examiners practiced applying pressure at a rate of approximately 1 kg/s. Standardized instructions were read to the participants: “The moment the pressure increases to a point where it first feels uncomfortable or painful, press and releases the button. This means the first onset of discomfort or pain and not the most pressure you can bear”. The PPT measurements were repeated three times with a pause of 10 s between the measurements. The average value of the three measurements was used as the individual's PPT of the lumbar multifidus muscles.

The peripheral blood was sampled between 8:00 a.m. and 9:00 a.m. during each clinic visit. The serum was harvested by centrifuging blood at 3,000 rpm (1,500 g) for 10 min and then stored at −80°C until assayed. The levels of TNF-α, IL-1β, and IL-6 in the serum were determined using ELISA kits (Solarbio Science & Technology Co. Ltd., China), and the PGE_2_ was determined using ELISA kits (Cusabio Biotech Co. Ltd., China). The procedures were performed according to the manufacturer's instructions. The specimens were examined using FLUOstar Omega (BMG Labtech, Germany). The levels of inflammatory cytokines were divided into low, moderate, and high levels according to tertiles of serum TNF-α, IL-1β, IL-6, and PGE_2_ in controls.

### Statistical analyses

Descriptive statistics were performed with STATA 13.0 (STATA Corp, TX, USA) to reveal the characteristics of participants by using count, percentage, and median with interquartile range (IQR). The normality of distribution was determined by the Shapiro-Wilk test. The Chi-square (χ^2^) test and the Wilcoxon test were used to evaluate the differences in demographic, occupational, and clinical characteristics between lower back MSDs cases and controls. Binary logistic regression was used to estimate odd ratios (ORs) with 95% confidence intervals (CI) for the associations of postural load and inflammatory cytokines with lower back MSDs. Linear regression was used to assess the association between postural load and inflammatory cytokines.

The following logistic equation can describe the mediation effect of binary outcomes. Where X is the independent variable, M is the mediator, and Y is the dependent variable. The coefficient c in equation (1) represents the total effect of X on Y when M is not considered, and e_1_ is the corresponding residual. The coefficient a in equation (2) represents the effect of X on M. The coefficient b in equation (3) represents the effect of M on Y after controlling for X, the coefficient c' represents the direct effect of X on Y after controlling for M, e_2_ and e_3_ represent the residuals of M and Y, respectively.


(1) $$\begin{array}{lll} Y^{\prime } & = & i_{1}+cX+e_{1} \end{array}$$

(2) $$\begin{array}{lll} M & = & i_{2}+aX+e_{2} \end{array}$$

(3) $$\begin{array}{lll} Y^{\prime \prime } & = & i_{3}+c^{\prime }X+bM+e_{3} \end{array}$$

(4) $$\begin{array}{lll} Y^{\prime } & = & LogitP\left(Y=1|X\right)=ln\frac{P\left(Y=1|X\right)}{P\left(Y=0|X\right)} \end{array}$$

(5) $$\begin{array}{lll} Y^{\prime \prime } & = & LogitP\left(Y=1|M,X\right)=ln\frac{P\left(Y=1|M,\;X\right)}{P\left(Y=0|M,X\right)} \end{array}$$

In the mediation model of continuous outcomes, the mediation effect is equal to ab or c-c'. However, for the mediation model of categorical X, continuous M and Y, the regression coefficients are not on the same scale, and a standardized regression coefficient conversion need to be carried out. The conversion method is as follows:


(6) $$\begin{array}{lll} a^{std} & = & a\cdot \frac{SD\left(X\right)}{SD\left(M\right)} \end{array}$$

(7) $$\begin{array}{lll} b^{std} & = & b\cdot \frac{SD\left(M\right)}{SD\left(Y^{\prime \prime }\right)} \end{array}$$

(8) $$\begin{array}{lll} c^{std} & = & c\cdot \frac{SD\left(X\right)}{SD\left(Y^{\prime }\right)} \end{array}$$

(9) $$\begin{array}{lll} c^{{}^{\prime }std} & = & c^{\prime }\cdot \frac{SD\left(X\right)}{SD\left(Y^{\prime \prime }\right)} \end{array}$$

(10) $$\begin{array}{lll} SD\left(Y^{\prime }\right) & = & \sqrt{c^{2}var\left(X\right)+\frac{\pi^{2}}{3}} \end{array}$$

(11) $$\begin{array}{lll} SD\left(Y”\right) & = & \sqrt{{c^{\prime }}^{2}var\left(X\right)+b^{2}var\left(M\right)+2c^{{}^{\prime }}bcov\left(X,M\right)+\frac{\pi^{2}}{3}} \end{array}$$

The standardized coefficients and the mediation effect were calculated using MPLUS 7.0 (Muthén and Muthén, CA, USA). The nonparametric percentile bootstrap method with deviation correction was adopted to test the significance of the mediation effect. The number of bootstrap operation samples was set to 1,000, and the confidence level and the confidence interval were taken as 95%.

## Results

### Demographic, occupational, and clinical characteristics between lower back MSDs cases and controls

As is seen in Table 1, the results showed that there was no significant difference between the cases and controls in gender, education, age, BMI, and physical exercise time per week (*P* > 0.05). In terms of occupational exposure, the hours spent per day working, the hours spent per day working in a sitting position, and the RULA score in the cases were higher than those in the controls (*P* < 0.05). Regarding clinical characteristics, PPTs and the level of PGE_2_ in the cases were significantly lower than those in the controls, while the levels of TNF-α, IL-1β, and IL-6 in the cases were significantly higher than those in the controls (*P* < 0.05).

**Table 1:** Demographic, occupational, and clinical characteristics of lower back MSDs cases and controls (*N* = 132).

**Items**	**Cases *(N* = 66)**	**Controls (*N* = 66)**	***P* ^c^**
Gender ^a^			1.000
-Male	19 (28.8)	19 (28.8)	
-Female	47 (71.2)	47 (71.2)	
Education ^a^			0.163
-Undergraduate	28 (42.4)	18 (27.3)	
-Master student	24 (36.4)	33 (50.0)	
-Doctoral student	14 (21.2)	15 (22.7)	
Age (years) ^b^	22 (23–25)	22.75 (23.5–25)	0.224
BMI (kg/m^2^) ^b^	20.89 (19.14–24.86)	21.06 (19.89–23.14)	0.314
Physical exercise time per week (minute) ^b^	45 (30–120)	60 (37.5–150)	0.467
Hours spent per day working (hours) ^b^	10 (8–12)	8 (7.75–10.25)	**0.014**
Hours spent per day working in a sitting position (hours) ^b^	9 (7.75–10)	8 (6–9)	**0.005**
Hours spent per day using computers (hours) ^b^	5 (4–8)	5 (3–8)	0.512
Hours spent per day using phones (hours) ^b^	6 (3–8)	6 (4–8)	0.702
RULA score			
-Continuous variable (1–7) ^b^	6 (4-−7)	4 (3–6)	**< 0.001**
-Categorical variable ^a^			**0.001**
-Low postural load (1–4)	18 (27.3)	38 (57.6)	
-Moderate postural load (5–6)	27 (40.9)	29 (28.8)	
-High postural load (7)	21 (31.8)	9 (13.6)	
PPTs (kg/cm^2^) ^b^	2.36 (1.85–3.62)	3.21 (2.74–4.36)	**< 0.001**
TNF-α (ng/L)			
-Continuous variable ^b^	5.69 (4.05–9.22)	4.65 (3.71–5.60)	**< 0.001**
-Categorical variable ^a^			**0.024**
-Low level	21 (31.8)	28 (42.4)	
-Moderate level	8 (12.1)	16 (24.3)	
-High level	37 (56.1)	22 (33.3)	
IL-1β (pg/L)			
-Continuous variable ^b^	133.00 (96.00–160.00)	91.00 (41.00–124.00)	**< 0.001**
-Categorical variable ^a^			**< 0.001**
-Low level	8 (12.1)	25 (37.9)	
-Moderate level	10 (15.2)	17 (25.7)	
-High level	48 (72.7)	24 (36.4)	
IL-6 (ng/L)			
-Continuous variable ^b^	4.16 (1.65–11.21)	1.71 (0.66–3.80)	**< 0.001**
-Categorical variable ^a^			**< 0.001**
-Low level	4 (6.1)	22 (33.3)	
-Moderate level	27 (40.9)	16 (24.3)	
-High level	35 (53.0)	28 (42.4)	
PGE_2_ (ng/L)			
-Continuous variable ^b^	70.06 (47.49–81.76)	125.62 (84.81–186.76)	**< 0.001**
-Categorical variable ^a^			**< 0.001**
-Low level	56 (84.8)	22 (33.3)	
-Moderate level	5 (7.6)	22 (33.3)	
-High level	5 (7.6)	22 (33.3)	

^a^ The χ^2^ test.

^b^ The Wilcoxon test.

^c^ Bold indicates P < 0.05.

### Association among postural load, inflammatory cytokines and lower back MSDs

**Figure 2:**
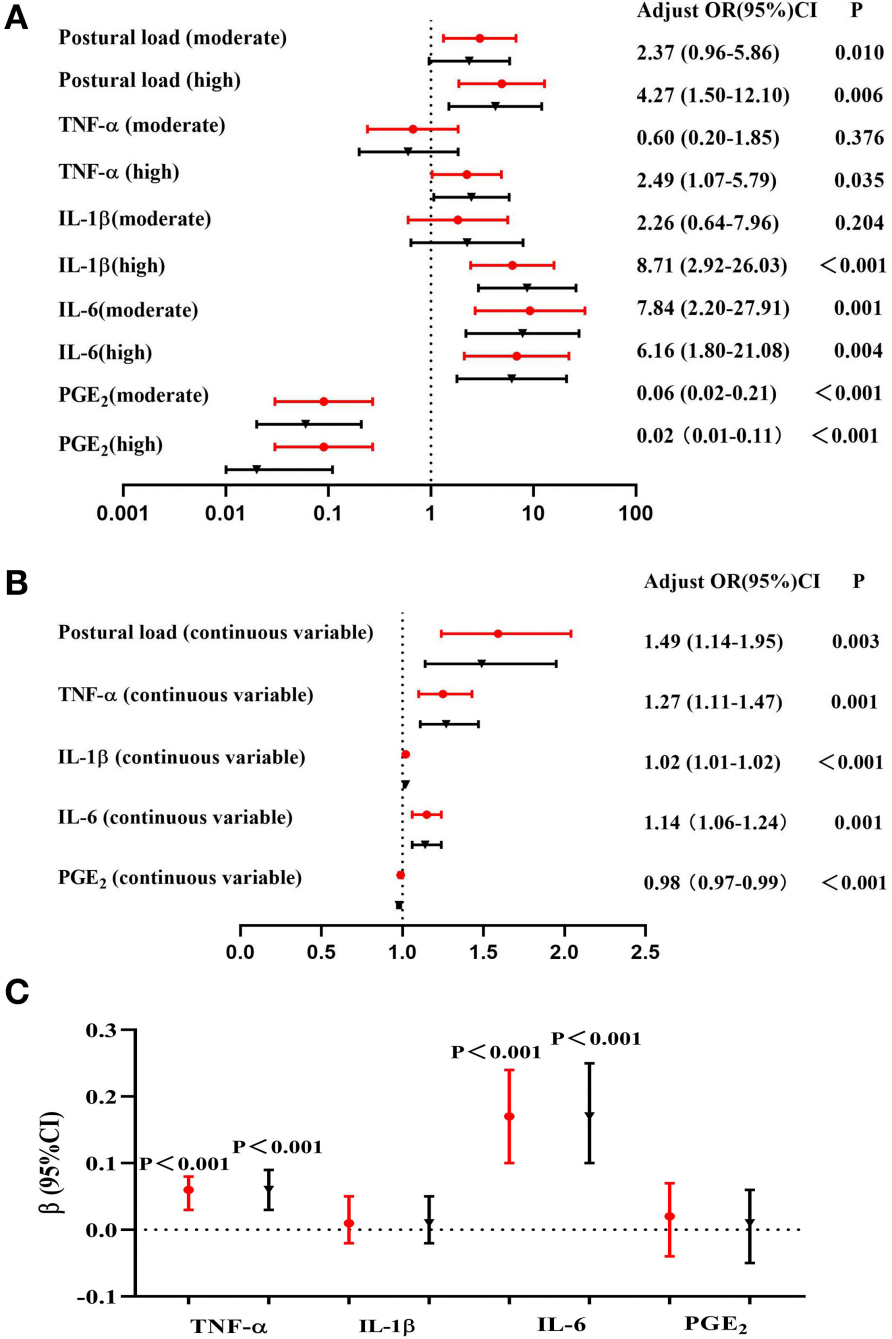
Association among postural load, inflammatory cytokines, and lower back MSDs (*N* = 132). **(A)** Association between postural load (categorical variable) and lower back MSDs, **(B)** Association between postural load (continuous variable) and lower back MSDs, **(C)** Association between postural load and inflammatory cytokines. The red circle indicates unadjusted models, and the black triangle indicates adjusted models (adjusted for gender, age, BMI, physical exercise time per week, hours spent per day working, hours spent per day working in a sitting position, hours spent per day using computers, and hours spent per day using phones).

The association of postural load and inflammatory cytokines with lower back MSDs are shown in Figures 2A,B. After adjusting for potential confounding factors, the results of logistic regression indicated that the risk of MSDs in the lower back increased by 1.49 times with an increase of one point of the RULA score. Taking the “low postural load” group as a reference, the risk of lower back MSDs in the “moderate postural load” and “high postural load” groups were 2.37 (95%CI: 0.96–5.86) and 4.27 (95%CI: 1.50–12.10), respectively. After adjusting for potential confounding factors, the results of logistic regression showed that with one unit increase in the levels of TNF-α, IL-1β, and IL-6 in serum, the risk of MSDs in the lower back increased by 1.27 times, 1.02 times and 1.14 times, respectively.

The association between postural load and inflammatory cytokines is shown in Figure 2C. The serum TNF-α, IL-1β, IL-6, and PGE_2_ levels of all participants were transformed to logarithmic variables. After adjusting for potential confounding factors, the results of linear regression showed that the levels of TNF-α and IL-6 were associated with postural load (β = 0.06, *P* < 0.001 and β = 0.17, *P* < 0.001, respectively).

### Mediation effect of inflammatory cytokines on the association between postural load and lower back MSDs

**Figure 3:**
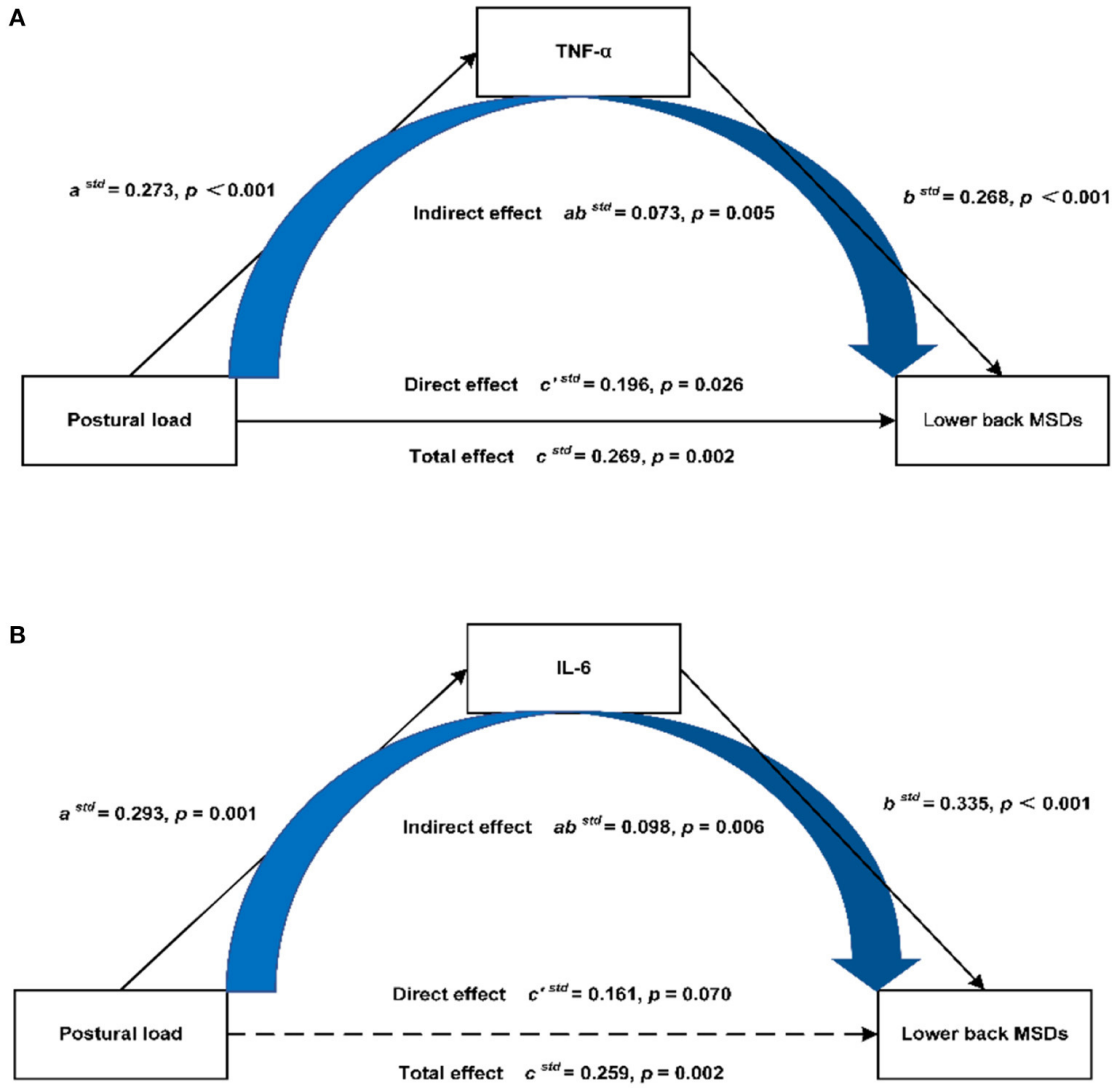
Mediation model of postural load on the lower back MSDs through inflammatory cytokines. **(A)** Mediation model of postural load on the lower back MSDs through TNF-α, **(B)** Mediation model of postural load on the lower back MSDs through IL-6.

After adjusting for gender, age, BMI, physical exercise time per week, hours spent per day working, hours spent per day working in a sitting position, hours spent per day using computers, and hours spent per day using phones, the results of mediation analysis showed that in the mediation model of TNF-α (Figure 3A), the total effect of postural load on the lower back MSDs was 0.269 (95%CI: 0.091–0.421), and the direct effect was 0.196 (95%CI: 0.018–0.360). The mediation effect of postural load on the lower back MSDs through TNF-α was 0.073 (95%CI: 0.025–0.128), accounting for 27.1% of the total effect. In the mediation model of IL-6 (Figure 3B), the total effect of postural load on the lower back MSDs was 0.259 (95%CI: 0.077–0.410), and the mediation effect of postural load on the lower back MSDs through IL-6 was 0.098 (95%CI: 0.041–0.179), accounting for 100% of the total effect.

## Discussion

In this study, we investigated the role of inflammation in the association between static postures exposure and MSDs based on a prospective nested case-control study. The major findings of our study include that: (a) the postural load, PPTs, and serum inflammatory cytokines were associated with lower back MSDs among university employees exposed to static postures, (b) the postural load was associated with inflammatory cytokines among university employees exposed to static postures, c) by the use of mediation analysis, we found that postural load affected lower back MSDs through inflammatory cytokines.

Our study showed a positive correlation between postural load and lower back MSDs among university employees exposed to static postures, which is consistent with previous studies (8, 22). A plausible hypothesis, which has been proposed previously and is supported by our findings, is that long-term static postures may change the internal biomechanical loading level or muscle tissue tolerance level by adjusting the physiologic reaction of the muscles, resulting in the internal biomechanical loading exceeding the tolerance of muscle tissues, which will cause tissue damages and lead to the occurrence of MSDs (23).

Another finding in our study was that lower back MSDs were associated with increased pain sensitivity. A previous systematic review and meta-analysis did not find such a significantly lower pain threshold among office workers with chronic neck pain when compared with healthy controls (24). But there is at least one study with findings in line with our study, that is a study of patients with carpal tunnel syndrome which showed a lower pain threshold than healthy controls (25). Our study also found musculoskeletal pain was associated with an increase in inflammatory cytokines. Previous studies have shown that inflammatory cytokines may be related to pain sensitivity, and inflammation may play an important role in the progression of pain (26, 27). For example, a few studies have indicated that higher plasma levels of pro-inflammatory cytokines TNF-α, IL-6, and IL-8 may be related to higher pain sensitivity (28, 29). The reason that inflammatory cytokines may affect the increase of pain sensitivity may be induced by the sensitization of peripheral nociceptors by pro-inflammatory mediators (30). It has also been shown that pro-inflammatory mediators, like cytokines signal the brain *via* humoral, vagal, and spinal pathways and induce symptoms of sickness behavior, including pain amplification (29). For example, TNF-α regulates the sensitivity of nociceptive neurons through potassium, sodium, and calcium ion channels, thus possibly participating in the pain pathway (31). IL-1β in addition to inducing inflammatory reaction, also directly activates nociceptors to generate action potentials and induce pain hypersensitivity (32). IL-6 may be involved in microglial and astrocytic activation and in the regulation of neuronal neuropeptide expression, and then induce pain hypersensitivity (33). PGE_2_ may cause pain by directly exciting nociceptive nociceptors and indirectly stimulating the release of pain-related neuropeptides, such as substance P and calcitonin gene-related peptides from nociceptors and their central and peripheral terminals (34).

Cytokines are proteins involved in many cellular processes, which are the chief stimulators of acute-phase-response proteins and contribute to the development and remediation of signs and symptoms of acute and chronic inflammation, such as the recruitment of immune cells. The increase of pro-inflammatory cytokines in serum indicates a chronic and/or systemic inflammatory reaction in the body. The cytokines TNF-α, IL-1, and IL-6 are intercellular signaling polypeptides produced by most injured cells and activated immune cells, including activated monocytes and macrophages (35). TNF-α and IL-1 can potently stimulate immune and stromal cell production of other cytokines and chemokines as well as most mechanisms of inflammation, including phagocyte proliferation and activation, adhesion, and angiogenesis (35). IL-6, a pleiotropic cytokine, has many pro-inflammatory effects that overlap with those of IL-1 and TNF-α. TNF-α and IL-1β are not influenced by the glycogen content of skeletal muscle and thus are not elevated in serum during exercise unless tissue damage has occurred (36). IL-6 is a tightly regulated cytokine normally not detectable in serum unless there is injury, infection, or cellular stress (12). PGE_2_ is a factor believed to cause vasodilation, edema, and increased release of inflammatory cytokines. When tissue injury occurs, PGE_2_ is produced by a large number of inflammatory cells in inflammatory tissue, which plays an essential role in the process of inflammation and pain.

Mediation analysis in the population provided an indication that postural load may affect the occurrence of MSDs through inflammatory cytokines such as TNF-a and IL-6. These new findings showed that there may be the participation of tissue inflammatory and systemic inflammation in the process of MSDs induced by static postures, which is in agreement with previous studies of repetitive tasks and exercise-induced skeletal muscle injuries (17, 18). Elevated systemic inflammation induces a pro-inflammatory response in tissues, such as muscles at the injury site (37). Conversely, inflamed tissues secrete inflammatory cytokines that contribute to systemic inflammation (38). This self-perpetuating cycle could underpin a chronically elevated inflammatory state with profound effects on the physiology and structure of musculoskeletal tissues, including connective tissues (39, 40).

To our best knowledge, this is an innovative study linking static postures and MSDs through a viewpoint of inflammatory pathways. The main strengths of our study are its prospective cohort-based, nested case-control design, which minimized selection bias and information bias. Our study provides evidence for the potential mechanism of increased MSDs risks associated with static postures exposure. Moreover, the increase of inflammatory cytokines may be a critical early event in MSDs progression and intervention target for the primary prevention of MSDs. Further studies are needed to investigate if preventing the occurrence of inflammation contributes to reducing the development of MSDs. However, several limitations should be noted. Firstly, variables may be influenced by subjective factors, and more objective methods such as clinical examination and special tests (such as surface electromyography) should be used in future research (41, 42). Secondly, the sample size of our study is limited, and it is quite advisable to include more occupational populations exposed to static postures to verify the results in future studies.

## Conclusions

Long-term static postures are closely related to the occurrence of MSDs through a viewpoint of inflammatory pathways, and low-level inflammation may be a critical early event in the generation of MSDs. Our findings may help bridge the gap of potential mechanisms linking static postures to increased risks of MSDs, and provide new evidence for targeted protection against the globally increasing MSDs. In addition, this study can likely contribute to the prevention of MSDs and improved health of the occupational population.

## Data availability statement

The raw data supporting the conclusions of this article will be made available by the authors, without undue reservation.

## Ethics statement

The studies involving human participants were reviewed and approved by Institutional Review Board of Peking University. The patients/participants provided their written informed consent to participate in this study.

## Author contributions

YD, YS, and LH were responsible for the study conceptualization, methodology development and validation, and study supervision. YD, PJ, XJ, NJ, and WH contributed to the conduction of population surveys. YD and PJ contributed to the preparation of samples and molecular biology experiments. YD contributed to the biostatistical analysis and interpretation and drafting of the manuscript. YD, XJ, NJ, WH, MF, and LY contributed to the critical revision of the manuscript for important intellectual content. All authors have read and approved the final version of the manuscript and agreed with the order of presentation of the authors.

## Funding

This study was supported by Foundation strengthening program of China (2020JCJQJJ238) and National Key Technologies Research and Development Program of China (2016YFC0801700).

## Conflict of interest

The authors declare that the research was conducted in the absence of any commercial or financial relationships that could be construed as a potential conflict of interest.

## Publisher's note

All claims expressed in this article are solely those of the authors and do not necessarily represent those of their affiliated organizations, or those of the publisher, the editors and the reviewers. Any product that may be evaluated in this article, or claim that may be made by its manufacturer, is not guaranteed or endorsed by the publisher.
